# A Modified Medical Education Research Study Quality Instrument (MMERSQI) developed by Delphi consensus

**DOI:** 10.1186/s12909-023-04033-6

**Published:** 2023-01-25

**Authors:** Mansour Al Asmri, M. Sayeed Haque, Jim Parle

**Affiliations:** 1 Clinical Skills Training Centre, King Fahad Specialist Hospital, Dammam, Saudi Arabia; 2 Institute of Applied Health Research, University of Birmingham, Birmingham, B15 2TT UK; 3 Institute of Clinical Sciences, College of Medical and Dental Sciences, University of Birmingham, Birmingham, B15 2TT UK

**Keywords:** Methodological quality, Quantitative studies, Delphi, Medical education, Quality instrument

## Abstract

**Background:**

The Medical Education Research Study Quality Instrument (MERSQI) is widely used to appraise the methodological quality of medical education studies. However, the MERSQI lacks some criteria which could facilitate better quality assessment. The objective of this study is to achieve consensus among experts on: (1) the MERSQI scoring system and the relative importance of each domain (2) modifications of the MERSQI.

**Method:**

A modified Delphi technique was used to achieve consensus among experts in the field of medical education. The initial item pool contained all items from MERSQI and items added in our previous published work. Each Delphi round comprised a questionnaire and, after the first iteration, an analysis and feedback report. We modified the quality instruments’ domains, items and sub-items and re-scored items/domains based on the Delphi panel feedback.

**Results:**

A total of 12 experts agreed to participate and were sent the first and second-round questionnaires. First round: 12 returned of which 11 contained analysable responses; second-round: 10 returned analysable responses. We started with seven domains with an initial item pool of 12 items and 38 sub-items. No change in the number of domains or items resulted from the Delphi process; however, the number of sub-items increased from 38 to 43 across the two Delphi rounds. In Delphi-2: eight respondents gave ‘study design’ the highest weighting while ‘setting’ was given the lowest weighting by all respondents. There was no change in the domains’ average weighting score and ranks between rounds.

**Conclusions:**

The final criteria list and the new domain weighting score of the Modified MERSQI (MMERSQI) was satisfactory to all respondents. We suggest that the MMERSQI, in building on the success of the MERSQI, may help further establish a reference standard of quality measures for many medical education studies.

**Supplementary Information:**

The online version contains supplementary material available at 10.1186/s12909-023-04033-6.

## Background

The Medical Education Research Study Quality Instrument (MERSQI) was introduced in 2007 to appraise the methodological quality of studies of medical education [1]. MERSQI evaluates the quality of the research itself rather than the quality of the reporting and the authors [1] excluded elements such as “importance of research questions” and “quality of conceptual frameworks”. MERSQI has been validated, gained acceptance and been widely used [2]. MERSQI contains ten items reflecting six domains: study design, sampling, type of data, validity of evaluation instrument, data analysis, and outcomes. All domains have the same maximum score of three; maximum score is 18. Previous research has established validity evidence for MERSQI including reliability and internal consistency, as well as relationship to other variables such as likelihood of publication, citation rate, and study funding [1–3]. Cook, DA and Reed, DA. [4] discussed and compared MERSQI with Newcastle–Ottawa scale-education method of evaluation and reported that MERSQI is a reliable tool for appraising the methodological quality of medical education research, however, it “lacks items on blinding and comparability of cohorts”. The limitations of MERSQI which are presented in our report have not been previously discussed or mentioned in the literature.

We argue that the existing instrument would be improved by adding or modifying the criteria to facilitate better quality assessment. We suggest that: (i) the risk of bias of randomised controlled trials should be considered [5]; (ii) participant characteristics should be included [6] (particularly in some domains such as teaching intimate examination skills); (iii) the robustness of objective data measurement required to discriminate learners’ level of mastery should be assessed, as per Miller’s pyramid [7]. Learning a skill goes through three stages [8]: cognitive (understanding), associative (practise), and autonomous (automatic). Thus, the learner could, for example, form a cognitive picture of the skill but lack the fundamentals and mechanics required to perform the skill. The cognitive framework is clearly a pre-requisite to enable practise. Similarly, to use Miller’s framework, learners progress through ‘knows’ to ‘knows how’ to ‘shows how’ to ‘does’ (by which Miller means performs in the real clinic as a practicing clinician). In assessing acquisition of skills, therefore, we argue that it is consistent with Miller’s pyramid to weight performance (e.g. in our context ‘high fidelity simulation’ which is the closest to actual performance in almost all these reported studies) above testing ‘on paper’ which clearly can only assess the cognitive imagining of a skill, not its performance as such. Furthermore, we argue that the impact that each of the six domains has on the quality of the study is not equal (indeed that this is clear a priori) and therefore, each domain should be weighted based on its impact on study quality, see for example Timmer et al. [9] who gave study design the highest score in the development and evaluation of a quality score for abstracts.

The purpose of this paper, therefore, is to report on our modification of the MERSQI utilising the Modified Delphi method [10]. We aimed to achieve consensus among experts on: (1) modifications of the MERSQI domains, items or sub-items (2) the MERSQI scoring system and weighting of each domain.

## Methods

### Research team

The research team consists of all the authors. The researchers have different backgrounds: clinical, academic, statistical, and simulation education.

### Selection of items

We included the initial pool of MERSQI items and included new items (Table 1) which we had developed in our previous work [11] to improve the granularity of the MERSQI. Based on the first modified MERSQI list, we created a Delphi questionnaire of 12 items under seven domains i.e. the original six domains plus a ‘settings’ domain. We used the Delphi method as it is implicitly based on both empirical evidence and personal opinion and allows conflicting scientific evidence to be addressed using quasi-anonymity of experts’ opinion [12–14]. We used the modified Delphi (i.e. utilising our previous work), because this method increases the response rate of the initial round [10]. Delphi rounds continue till sufficient consensus is reached (consensus is defined as general agreement of a substantial majority [12], please see procedure in the methods section for more details). Expert panel members were given the opportunity, in each round, to add items, to suggest rewording of items, to score items, and to weight the seven domains, see for example Timmer et al. [9] (Additional file 1).

**Table 1:** Showing original MERSQI and first Modification of the MERSQI, showing the new items used in Delphi-1

A. Original MERSQI Item	Domain	B. Modified MERSQI Item
1. Study design	Study design	1. Study design
Single group cross-sectional or single group post-test only		a. Single group cross-sectional or single group post-test only
Single group pre-test & post-test		b. Single group pre-test & post-test
Nonrandomized, 2 groups		c. Nonrandomised, 2 groups
Randomized controlled trial		d. *Randomised controlled trial with high risk bias*^a^
		e. *Randomised controlled trial with moderate risk bias*^a^
		f. *Randomised controlled trial with low risk bias*^a^
2. Institutions studied: *(Pls. select one)*	Sampling	1. *Is there a power calculation for sample size?*
1		2. *Are detailed participant characteristics for each arm reported?*
2		3. Response rate, %: (Pls. select one)
> 2		
3. Response rate, %: *(Pls. select one)*		
Not applicable		a. Not applicable
< 50 or not reported		b. < 50 or not reported
50‐74		c. 50‐74
> 75		d. > 75
	*Setting*	4. Institutions studied: *(Pls. select one)*
		a. Single centre
		b. Multi centre
4. Type of data	Type of data	5. Type of data
Assessment by participants		Assessment by participants
Objective measurement		Objective measurement (*Pls. select one)*
		a. *Knowledge test (e.g. recall type questions)*
		b. *Applied knowledge test (e.g. analysis and problem-solving type questions)*
		c. *Skills*
5. Internal structure:	Validity of evaluation instrument	6. Internal structure:
a. Not applicable		a. Not applicable
b. Not reported		b. Not reported
c. Reported		c. Reported
6. Content:		7. Content:
a. Not applicable		a. Not applicable
b. Not reported		b. Not reported
c. Reported		c. Reported
7. Relationships to other variables:		8. Relationships to other variables:
a. Not applicable		a. Not applicable
b. Not reported		b. Not reported
c. Reported		c. Reported
8. Appropriateness of analysis:	Data analysis	9. Appropriateness of analysis:
a. Inappropriate for study design or type of data		a. Inappropriate for study design or type of data
b. Appropriate for study design, type of data		b. Appropriate for study design, type of data
9. Complexity of analysis:		10. Complexity of analysis: (*Pls. select one)*
a. Descriptive analysis only		a. Descriptive analysis only
b. Beyond descriptive analysis		b. *Simple inferential statistics*
		c. *Modelling and more complex analysis*
10. Outcomes	Outcomes	11. Outcomes
Satisfaction, attitudes, perceptions, opinions, general facts		Satisfaction, attitudes, perceptions, opinions, general facts
Knowledge, skills		Knowledge, skills measured by: (*Pls. select one)*
Behaviours		a. *Low fidelity simulation or paper-based assessments*
Patient/health care outcome		b. *High fidelity simulation*
		Behaviours *in clinical environment*
		Patient/health care outcome

a Risk of bias judgment based on: sequence generation, blinding & allocation concealment. For more details, please see Additional file 2 Cochrane Risk of Bias Tool for Randomized Controlled Trials

### Selection of expert panel

Potential panel members were identified based on our knowledge of their fields of interest and published work in medical education research. We identified 22 potential respondents who were approached by email. There was no response from 7 and 3 declined. All twelve respondents were experts in medical education: one Clinical Outcomes Assessment Consultant, one Associate Professor of Education, one Professor of Health Sciences and Medical Education, and one Professor of Clinical Communication as well as eight medical academics: two Professors of Medical Education, two Associate Professors of Medical Education, one Professor of General Practice, one Professor of Simulation Education, one Professor of Anaesthetics, and one Professor of Clinical Epidemiology.

### Procedure

Questionnaires were distributed to panel members by emails. In the first round (Delphi-1) we requested respondents to (i) give a score that reflected research quality for items and/or sub-items (in case the item has multiple choices) within each domain; on a scale of one to ten, ten being the highest (ii) indicate whether there should be any additional items, or modifications to the existing ones (iii) estimate the weighting for each domain out of 100 available points to be allocated across the domains. For Delphi-2, a feedback report was prepared and shared anonymously with respondents, summarising responses with additional items included as recommended in Delphi-1. Items or sub-items were added, removed, or modified if eight or more out of twelve panellists agreed. We considered consensus had been achieved when the agreement rate reaches 70% or more amongst respondents [15]. In the Delphi-1free text feedback it was clear that the respondents had different interpretations of high and low simulation fidelity, as is common in the literature [16]. Subsequently, in Delphi-2 we provided them with a clear definition of high fidelity, which we defined as “the ability of the simulated training to provide a true representation of intended learning goals”. Respondents were also provided with their previous scores plus the mean score of other respondents (anonymised) on each item from the previous round. They were asked to score any new items and re-evaluate their previous scores, bearing in mind the scores given by the rest of the panel, altering their score if they wished.

This procedure (Delphi rounds) is ended if a general consensus is achieved by visual inspection in all the domains with respect to domain weighting or ranks between two subsequent Delphi rounds [17].

The University of Birmingham Research Ethics Committee (reference number ERN_20-0728) approved this study.

## Results

### Delphi round one

All 12 experts (7 male, and 5 female) returned the questionnaires. Eight respondents were from the UK and four were from outside the UK; respondents were from nine different institutions. Unfortunately, one of the questionnaires was returned unusable (mostly blank) and therefore was excluded from analysis.

Respondents suggested five sub-items to be added (**Bold** and *Italic* in Table 2). The ‘study design’ domain was given the highest weighting by eight (73%) respondents although five of these eight respondents scored study design equal highest with another domain. Two (18%) respondents gave data analysis the highest weighting and one (9%) scored outcomes highest.

**Table 2:** The final Modified MERSQI; items after Deplhi-1, scores after Delphi-2

Domain	MMERSQI Item	Points	Score		
			Each	Total	Max
Study design	1. Study design: (Pls. select one)				
	a. Single group cross-sectional or single group post-test only	7			23
	b. Single group pre-test & post-test	9			
	c. Nonrandomised, 2 groups	10			
	d. Randomised controlled trial with high risk bias^a^	11			
	e. Randomised controlled trial with moderate risk bias^a^	16			
	f. Randomised controlled trial with low risk bias^a^	23			
Sampling	2. Is there a power calculation *(****sufficient statistical power****)* for sample size?				10
	a. No	0			
	b. Yes	3			
	3. Are detailed participant characteristics for each arm reported?				
	a. No	0			
	b. Yes	3			
	4. Response rate, %: (Pls. select one)				
	a. Not reported	0.5			
	b. < 50	1			
	c. 50‐74	2			
	d. > 75	4			
*Setting*	5. Institutions studied: *(Pls. select one)*				8
	a. Single centre	5			
	**b. *Multi centre no further specification***	5			
	**c. *Multi centre with specification but not appropriate / balanced / complementary***	5			
	**d. *Multi centre with appropriate and balanced / complementary***	8			
Type of data	6. Type of data				11
	Assessment by participants	4			
	Objective measurement (*Pls. select one)*				
	a. Knowledge test (e.g. recall type questions)	6			
	b. Applied knowledge test (e.g. analysis and problem-solving type questions)	8			
	c. Skills	11			
Validity of evaluation instrument	7. Internal structure:				15
	a. Not applicable				
	b. Not reported	0			
	c. Reported	5			
	8. Content:				
	a. Not applicable				
	b. Not reported	0			
	c. Reported	5			
	9. Relationships to other variables:				
	a. Not applicable				
	b. Not reported	0			
	a. Reported	5			
Data analysis	10. Appropriateness of analysis:				17
	a. Inappropriate for study design or type of data	0			
	b. Appropriate for study design, type of data	9			
	11. Complexity of analysis (***if appropriate for study design***): *(Pls. select one)*				
	a. Descriptive analysis only	4			
	b. Simple inferential statistics	4			
	c. Modelling and more complex analysis	8			
Outcomes	12. Outcomes				
	Satisfaction, attitudes, perceptions, opinions, general facts	7			16
	***Knowledge, measured by: (Pls. select one)***				
	**a. *Low fidelity simulation or paper-based assessments***	9			
	**b. *High fidelity simulation***	12			
	***Skills measured by:***				
	**a. *Low fidelity simulation or paper-based assessments***	8			
	**b. *High fidelity simulation***	12			
	Behaviours in clinical environment	13			
	Patient/health care outcome	16			
Total Possible score				Min 23.5	Max 100

a Risk of bias judgment based on: sequence generation, blinding & allocation concealment. For more details, please see Additional file 2 Cochrane Risk of Bias Tool for Randomized Controlled Trials

### Delphi round two

For Delphi-2, 12 questionnaires were distributed and 10 were returned. Of the two non-responders, one had not responded to the first round. Only two respondents modified their distribution of score weighting between the domains. Eight (80%) respondents gave ‘study design’ the highest weighting (average 23 percentage points) and ‘setting’ was given the lowest weighting by all respondents (average 8 percentage points) (Table 3). Five of the eight respondents weighted study design equally with another domain. The domains weighted equally to the study design domain were outcomes (by three respondents), evaluation instrument validity domain (by one respondent) and data analysis domain (by one respondent). As can be seen in Table 3 there is general consensus in all these domains. There was no change in domain average weighting or ranks between Delphi-1 and Delphi-2. Therefore, we ended the Delphi rounds.

**Table 3:** Domains weighting score (to sum up 100)

Respondents No		Study design	Sampling	Setting	Type of data	Evaluation instrument validity	Data analysis	Outcomes
Respondents did not change scores between rounds	1	**25**	**10**	**5**	**0**	**10**	**35**	**15**
	2	**25**	**10**	**5**	**10**	**20**	**10**	**20**
	3	**20**	**10**	**10**	**15**	**15**	**10**	**20**
	4	**20**	**14**	**7**	**15**	**20**	**14**	**10**
	5	**15**	**15**	**10**	**10**	**15**	**20**	**15**
	6	**30**	**10**	**5**	**15**	**20**	**10**	**10**
	7	**30**	**5**	**5**	**10**	**10**	**30**	**10**
	8	**20**	**10**	**10**	**10**	**20**	**10**	**20**
Respondents changed their scoors	9	10^a^	**10**	15 ^a^	**10**	**15**	**15**	25 ^a^
		20^b^		10 ^b^				20 ^b^
	10	**25**	**10**	**10**	**10**	**10**	10 ^a^	25 ^a^
							15 ^b^	20 ^b^
Respondent no response round 2	11	30 ^a^	10 ^a^	10 ^a^	20 ^a^	5 ^a^	20 ^a^	5 ^a^
		NR ^b^	NR ^b^	NR ^b^	NR ^b^	NR ^b^	NR ^b^	NR ^b^
Delphi-1 Mean		23	10	8	11	15	17	16
RANK		1	6	7	5	4	2	3
Delphi-2 Mean		23	10	8	11	15	17	16
Standard errors (SE) of Mean		1.5	0.8	0.8	1.4	1.4	2.8	1.5
RANK		1	6	7	5	4	2	3
change in rank?		0.0	0.0	0.0	0.0	0.0	0.0	0.0

*NR* No response

a Delphi-1 score

b Delphi-2 score; Bold = Delphi 1&2 score (no change in score)

We used the average weighting score (out of 100) to determine the weighting of each domain. Thus, for example, ‘study design’ received the average weight of 23 out of the 100 points available and so each sub-item within that domain had the ‘possibility’ of scoring the full 23 points. We used the score out of ten which had been given by respondents for each sub-item to then allocate a proportion (in this example up to a maximum of 23 points) to each sub-item in this domain. Thus, for example, the sub-item ‘single group cross-sectional or single group post-test only’ scored 3/10 and was thus allocated three tenths of the available 23 points for that domain (i.e. 7). In contrast, ‘Randomised controlled trial with low-risk bias’ scored 9/10 and was therefore allocated 21 points (i.e. 90% of the domain weighting (23)). For simplicity, we rounded up the points for the item which achieved the highest points in each domain so that the overall total had at least the possibility of achieving 100. For domains where more than one sub-item could be scored, we used the highest scoring item. For example, in the data analysis domain, the maximum possible score is 17. This domain has two items and each item has multiple sub-items. If scoring a paper containing both simple inferential statistics and modelling, we use the highest scoring item, and thus 8 points (for modelling) are awarded rather than 4 points (for simple inferential statistics). The final quality criteria list is shown in Table 2.

### Summary and discussions

A group of respondents with known relevant expertise [11] participated in two Delphi rounds to achieve consensus on MMERSQI. We derived our MMERSQI from the original MERSQI with the addition of items developed by the research team and which have been supplemented and assessed through a Delphi process.

After two rounds, there was a clear consensus that some domains have significantly more importance in determining educational research quality. It is of course possible that a different expert panel would have given different results, but our panel consisted of a wide range of people from different perspectives who were all experts in medical education. However, the standard errors (SE) of the mean are very small, thus the probable scores that may be given by other panels would most likely not vary much from the scores we got from this panel.

The learning effectiveness of simulation-based medical education is well-established in the literature [18, 19]. Of course, simulation-based medical education cannot replace but can support and supplement clinical placement in terms of effectiveness, self-confidence, and preparation for clinical practice [20]. Surprisingly small differences were found between the points given by the Delphi panel to high fidelity simulation (accuracy of simulation) (12 points) compared to the clinical environment (13 points).This is consistent with report from Quail et al. [21] that learning communication skills in a brief placement in virtual, standardised, or traditional learning environments achieved the same outcomes in knowledge and confidence.

The fidelity of the training has to be high for all types of learners and constant all the time but focus must be shifted from the *appearance* to the accuracy of stimulus, information processing and response in a certain situation. If a learner has learned a skill incorrectly for the first time, it appears, a priori, that performance may be hindered even with further training [22, 23]. On the other hand, the difficulty / simplicity level of the simulated training should match the learner level to improve engagement in learning [24]. As Vygotsky [25] says, skills development takes place in the zone of the learner being able to solve a problem independently or with help of an expert as described by the concept of the zone of proximal development. The most important issue therefore is the ability of the simulation to achieve the intended transferable learning goals.

## Conclusion

The Delphi process achieved consensus on the MMERSQI. Respondents achieved consensus that the domain weighting should not be equal and that some domains have more importance than others. We suggest that the MMERSQI, in building on the success of the MERSQI, may help further establish a minimum reference standard of quality measures for medical education studies. The validity of this criteria list and scoring system will have to be further evaluated over time.

## Supplementary Information

**Additional file 1:** Quality scoring instrument.

**Additional file 2:** Cochrane Risk of Bias Tool for Randomized Controlled Trials.

**Additional file 3.** A comparison of the original MERSQI and MMERSQI scores on a sample of 12 studies.

## Data Availability

The datasets used and/or analysed during the current study are available in an anonymised format from the corresponding author on reasonable request.

## Abbreviations

MERSQI: Medical Education Research Study Quality Instrument
MMERSQI: Modified Medical Education Research Study Quality Instrument
