# Supplementary material for: A Modified Medical Education Research Study Quality Instrument (MMERSQI) developed by Delphi consensus
Source: BMC Med Educ. 2023 Jan 25;23:63. doi: 10.1186/s12909-023-04033-6 (PMC9878889; doi:10.1186/s12909-023-04033-6)
Supplement: Supplementary file 1 — Additional file 1: Quality scoring instrument. [file 12909_2023_4033_MOESM1_ESM.doc]

Appendix 1: Quality scoring instrument

Study design Points

Human

*interventional:*

- parallel controlled trial 4 Randomization reported? (+1)
- cross over trial 3
- time series trial (before-after) 2
- non-concurrent / historic controls 2
- natural experiment 2

*observational*

- cohort, prospective 4
- cohort, retrospective 3
- cross-sectional 3
- case-control 3
- descript., case report/series 1

Basic Science

*interventional:*

- parallel controlled 4
- cross over trial 3
- set of before-after trials 3
- before-after, no controls 2

*observational*

- comparative 3
- case series 1

Other:

- meta analysis 2
- instrument validation 1
- literature review 1
- other: 0
- don’t know / not sure 0

**Scoring:**

For each applicable item, 0-2 points are awarded (2 if fully met, 1 if partially met, 0 if not met). In addition, points are awarded based on the study design and based on whether randomization was present (0-5). The maximum possible total is (19 * 2) + 5 = 43. For each item which is not applicable, such as blinding of subjects in basic science research, 2 points are subtracted from 43, resulting in the total possible score. The summary score is calculated by dividing the total score achieved by the total possible score.

| **Quality assessment** | yes | partial | no | n/a |
| --- | --- | --- | --- | --- |
| 1. Question / objective sufficiently described? |  |  |  |  |
| 1. Design evident and appropriate to answer study question? |  |  |  |  |
| 1. Subject characteristics sufficiently described? |  |  |  |  |
| 1. Subjects appropriate to the study question? |  |  |  |  |
| 1. Controls used and appropriate? **(if no control, check no)** |  |  |  |  |
| 1. Method of subject selection described and appropriate? |  |  |  |  |
| 1. If random allocation to treatment groups was possible, is it described? (if not possible, check n/a) |  |  |  |  |
| 1. If blinding of investigators to intervention was possible, is it reported? (If not possible, n/a) |  |  |  |  |
| 1. If blinding of subjects to intervention was possible, is it reported? (If not possible, n/a)^1^ |  |  |  |  |
| 1. Outcome measure well defined and robust to measurement bias? Means of assessment reported? |  |  |  |  |
| 1. Confounding accounted for? |  |  |  |  |
| 1. Sample size adequate? |  |  |  |  |
| 1. Post hoc power calculations or confidence intervals reported for statistically non significant results? |  |  |  |  |
| 1. Statistical analyses appropriate? |  |  |  |  |
| 1. Statistical tests stated? |  |  |  |  |
| 1. Exact p-values or confidence intervals stated? |  |  |  |  |
| 1. Attrition of subjects and reason for attrition recorded? |  |  |  |  |
| 1. Results reported in sufficient detail? |  |  |  |  |
| 1. Do the results support the conclusions? |  |  |  |  |
| Sum (items 1-19) |  |  |  |  |

How to calculate a summary score:

The summary score is calculated as the ratio of the points awarded to the maximum possible score. The maximum possible score depends on the number of items that are applicable based on the type of research. There are more applicable items for more sophisticated designs, such as controlled clinical trials than for simpler studies, such as case reports. For each quality assessment item, a maximum of 2 points is awarded (0 if not met, 1 if partially met, 2 if fully met), resulting in a maximum of 38 if all quality assessment items are applicable and fully met, plus 5 points for design. Points awarded based on design serve to counterbalance inequalitities arising from the applicability of the various items. E.g. for randomized parallel controlled trials, to get a full score (2 points) for control for confounding, beside randomization, a statement on the comparability of relevant prognostic factors between the groups, or else, additional measures of control are needed. In contrast, in cross over trials, comparability of confounding factors is assumed to be present. Consequently, if weighted equally, cross over trials would tend to score better, irrespective of quality. Thus, the points for design do not constitute a value judgment of design. Rather, the appropriateness of the design was considered to depend on the nature of the research question and was assessed in item 2 of the quality instrument, “description and appropriateness of the study design”. Also, there is a deduction of 2 points for any uncontrolled design (item 5).

Examples:

a) All 19 items apply to a controlled trial with negative results if double blinding and randomization are feasible, resulting in a maximum possible sum of points of 43 (2*19 + 5 for design). In a report, randomization is mentioned, but the method of randomization (item 7), and power calculations or confidence intervals (item 13) are not provided resulting in a deduction of 4 points. All other items are fully met. The sum of points is 39 (43-4), resulting in a summary score of 39/43 = 0.91.

b) For a case series, the items 7 to 17 (= 11 items) are not applicable, resulting in a maximum possible total of 21 generated from 8 applicable items plus 5 points (design). In a sample abstract, 5 of 8 required items are fully met (5*2 = 10 points), but the rationale for presenting the series is not entirely clear (item 1, partially met = 1 point). There is a further deduction for lack of controls (item 5 = 0 point). The study design is given 1 point (out of 5). Thus, the total is 10+1+1+0 = 12. The summary score is then calculated as 12/21 = 0.57.
