# Supplementary material for: A Modified Medical Education Research Study Quality Instrument (MMERSQI) developed by Delphi consensus
Source: BMC Med Educ. 2023 Jan 25;23:63. doi: 10.1186/s12909-023-04033-6 (PMC9878889; doi:10.1186/s12909-023-04033-6)
Supplement: Supplementary file 2 — Additional file 2: Cochrane Risk of Bias Tool for Randomized Controlled Trials. [file 12909_2023_4033_MOESM2_ESM.docx]

**Appendices**

| **Appendix - 1 Cochrane Risk of Bias Tool for Randomized Controlled Trials** | |
| --- | --- |
| **RANDOM SEQUENCE GENERATION** | |
| **Selection bias (biased allocation to interventions) due to inadequate generation of a randomised sequence.** | |
| Criteria for a judgment of ‘Low risk’ of bias. | The investigators describe a random component in the sequence generation process such as: •Referring to a random number table; •Using a computer random number generator; •Coin tossing; •Shuffling cards or envelopes; •Throwing dice; •Drawing of lots; |
| Criteria for the judgment of ‘High risk’ of bias. | The investigators describe a non-random component in the sequence generation process. Usually, the description would involve some systematic, non-random approach, for example: •Sequence generated by odd or even date of birth; •Sequence generated by some rule based on date (or day) of admission; •Sequence generated by some rule based on hospital or clinic record number. Other non-random approaches happen much less frequently than the systematic approaches mentioned above and tend to be obvious. They usually involve judgement or some method of non-random categorization of participants, for example: •Allocation by judgement of the clinician; •Allocation by preference of the participant; •Allocation based on the results of a laboratory test or a series of tests; •Allocation by availability of the intervention. |

| **ALLOCATION CONCEALMENT Selection bias (biased allocation to interventions) due to inadequate concealment of allocations prior to assignment.** | |
| --- | --- |
| Criteria for a judgment of ‘Low risk’ of bias. | Participants and investigators enrolling participants could not foresee assignment because one of the following, or an equivalent method, was used to conceal allocation: •Central allocation (including telephone, web-based and pharmacy-controlled randomization); •Sequentially numbered drug containers of identical appearance; •Sequentially numbered, opaque, sealed envelopes. |
| Criteria for the judgment of ‘High risk’ of bias. | Participants or investigators enrolling participants could possibly foresee assignments and thus introduce selection bias, such as allocation based on: •Using an open random allocation schedule (e.g. a list of random numbers); •Assignment envelopes were used without appropriate safeguards (e.g. if envelopes were unsealed or non-opaque or not sequentially numbered); •Alternation or rotation;•Date of birth; •Case record number; •Any other explicitly unconcealed procedure. |

| **BLINDING OF OUTCOME ASSESSMENT Detection bias due to knowledge of the allocated interventions by outcome assessors.** | |  |
| --- | --- | --- |
| Criteria for a judgment of ‘Low risk’ of bias. | Any one of the following: •No blinding of outcome assessment, but the review authors judge that the outcome measurement is not likely to be influenced by lack of blinding; •Blinding of outcome assessment ensured, and unlikely that the blinding could have been broken. | |
| Criteria for the judgment of ‘High risk’ of bias. | Any one of the following: •No blinding of outcome assessment, and the outcome measurement is likely to be influenced by lack of blinding; •Blinding of outcome assessment, but likely that the blinding could have been broken, and the outcome measurement is likely to be influenced by lack of blinding. | |
| Criteria for the judgment of ‘Unclear risk’ of bias. | Any one of the following: •Insufficient information to permit judgment of ‘Low risk’ or ‘High risk’; •The study did not address this outcome. | |
