# Supplementary material for: A Modified Medical Education Research Study Quality Instrument (MMERSQI) developed by Delphi consensus
Source: BMC Med Educ. 2023 Jan 25;23:63. doi: 10.1186/s12909-023-04033-6 (PMC9878889; doi:10.1186/s12909-023-04033-6)
Supplement: Supplementary file 3 — Additional file 3. A comparison of the original MERSQI and MMERSQI scores on a sample of 12 studies. [file 12909_2023_4033_MOESM3_ESM.docx]

A comparison of the original MERSQI and MMERSQI scores on a sample of 12 studies

| No. | Author (year) | Mean (MA&JP)  original  MERSQI | RANK | Mean (MA&JP)  MMERSQI | rank | Change in rank |
| --- | --- | --- | --- | --- | --- | --- |
|  | Robb et al. | 69% | 1 | 57% | 2 | -1 |
|  | Carrasco et al. | 67% | 2 | 60% | 1 | +1 |
|  | Gerling, Rigsbee, Childress, and Martin | 65% | 3 | 56% | 4 | -1 |
|  | Hendrickx et al. | 64% | 4 | 53% | 6 | -2 |
|  | Popadiuk et al. | 62.5% | 5 | 56.5% | 3 | +2 |
|  | Siebeck et al. | 61% | 6 | 56% | 4 | +2 |
|  | Rodriguez-Diez et al. | 50% | 7 | 44.5% | 10 | -3 |
|  | Isherwood et al. | 47% | 8 | 49.5% | 7 | +1 |
|  | Pugh et al. | 46.5% | 9 | 45% | 9 | 0 |
|  | Hegele et al. | 44.5% | 10 | 47.5% | 8 | +2 |
|  | Stolarek | 36% | 11 | 41.5% | 11 | 0 |
|  | Cohen et al. | 32% | 12 | 37.5% | 12 | 0 |

1. Robb A, Kopper R, Ambani R, et al. Leveraging virtual humans to effectively prepare learners for stressful interpersonal experiences. IEEE Trans Vis Comput Graph 2013;19:662–70.
2. Carrasco J, Gómez E, García J, et al. Impact of the use of simulators on the mental workload and confidence in a digital rectal examination and bladder catheterization workshop. Arch Esp Urol 2018;71:537–42.
3. Gerling GJ, Rigsbee S, Childress RM, et al. The design and evaluation of a computerized and physical simulator for training clinical prostate exams. IEEE Trans Syst Man Cybern Part A 2009;39:388–403.
4. Hendrickx K, De Winter B, Tjalma W, et al. Learning intimate examinations with simulated patients: the evaluation of medical students’ performance. Med Teach 2009;31:e139-e147.
5. Popadiuk C, Pottle M, Curran V. Teaching digital rectal examinations to medical students: an evaluation study of teaching methods. Acad Med 2002;77:1140–6.
6. Siebeck M, Schwald B, Frey C, et al. Teaching the rectal examination with simulations: effects on knowledge acquisition and inhibition. Med Edu 2011;45:1025–31.
7. Rodriguez-Diez MC, Diez N, Merino I, et al. Simulators help improve student conidence to acquire skills in urology. Actas Urol Esp 2014;38:367–72.
8. Isherwood J, Ashkir Z, Panteleimonitis S, et al. Teaching digital rectal examination to medical students using a structured workshop - a point in the right direction? J Surg Educ 2012;70:254–7.
9. Pugh CM, Iannitelli KB, Rooney D, et al. Use of mannequin-based simulation to decrease student anxiety prior to interacting with male teaching associates. Teach Learn Med 2012;24:122–7.
10. Hegele A, Heers H, Bruening F, et al. How can young academics be recruited? Acceptance and effects of urological practice-oriented training. Urologe 2014;53:236–40.
11. Stolarek I. Procedural and examination skills of first-year house surgeons: a comparison of a simulation workshop versus 6 months of clinical ward experience alone. N Z Med J 2007;120:U2516–U2516.
12. Cohen E, Ononye C, Salud J, et al. Use of simulation to understand the effects of task complexity and time away on clinical confidence. Stud Health Technol Inform 2013;184:92–5.
